# Novel strategies in tendon and ligament tissue engineering: Advanced biomaterials and regeneration motifs

**DOI:** 10.1186/1758-2555-2-20

**Published:** 2010-08-20

**Authors:** Catherine K Kuo, Joseph E Marturano, Rocky S Tuan

**Affiliations:** 1 Department of Biomedical Engineering, Tufts University, Medford, MA 02155, USA; 2 Center for Cellular and Molecular Engineering, Department of Orthopaedic Surgery, University of Pittsburgh School of Medicine, Pittsburgh, PA 15219, USA

## Abstract

Tendon and ligaments have poor healing capacity and when injured often require surgical intervention. Tissue replacement via autografts and allografts are non-ideal strategies that can lead to future problems. As an alternative, scaffold-based tissue engineering strategies are being pursued. In this review, we describe design considerations and major recent advancements of scaffolds for tendon/ligament engineering. Specifically, we outline native tendon/ligament characteristics critical for design parameters and outcome measures, and introduce synthetic and naturally-derived biomaterials used in tendon/ligament scaffolds. We will describe applications of these biomaterials in advanced tendon/ligament engineering strategies including the utility of scaffold functionalization, cyclic strain, growth factors, and interface considerations. The goal of this review is to compile and interpret the important findings of recent tendon/ligament engineering research in an effort towards the advancement of regenerative strategies.

## Review

The primary role of tendon and ligament is to transfer forces between musculoskeletal tissues. Adult tendon and ligament have relatively low oxygen and nutrient requirements, low cell density, and poor regenerative capacity, yet they experience some of the highest mechanical loads in the body. When these loads exceed a critical threshold that causes permanent tissue damage, impaired function and mobility will result. For the repair of both tissues, given their very low self-regenerative capacity, typically the only recourse is surgical intervention. Current surgical reparative techniques rely on tissue replacement with auto- or allografts, and are often accompanied with additional problems such as donor site morbidity, pain and graft failure. A more ideal solution would be to fully restore the tendon or ligament tissue to its pre-injured state. This is the promise of tissue engineering, a field which aims to incorporate specific cell types into a biodegradable scaffold which when implanted will gradually regenerate into a tissue that closely resembles the original tissue and restores functionality. In this review, we outline the state of the art of tendon and ligament (T/L) tissue engineering. Relatively little is known about these tissues as compared to other musculoskeletal tissues such as bone, cartilage and muscle, though recent studies have made significant advances, and distinct biological differences between tendon and ligament are beginning to be recognized [1-3]. However, from an engineering standpoint these tissues are functionally similar, and thus as reviewed here, tissue engineering efforts commonly refer to these tissues interchangeably. This review will discuss biomaterial selection and functionalization, scaffold design, cellular activities critical for tissue function, and recent outcomes of long-term implantation studies in animal models.

### Native tendon and ligament macromolecular composition and microenvironment

T/L properties that tissue engineers often try to mimic are the biochemical composition and structure, cell population, and mechanical properties of a native tissue. T/L obtain their tensile strength and elasticity from the molecular building block of their structure: triple-helical type I collagen molecules. A dimensional hierarchy exists where collagen molecules (~1 nm diameter) organize into parallel nanoscale fibrils, and subsequently into microscale fibers, both highly aligned along the major axis of the T/L unit. Fibril diameters range from 50-100 nm during embryonic development [4] and can reach as large as 280 nm in adults [5], and show a characteristic 67 nm lateral D-banding pattern coincident with molecular gaps [6]. Collagen type I fibrils account for the majority (~60%) of T/L dry mass and are surrounded by other proteins, including collagen types III, IV, V, and VI, and small-leucine rich proteins (SLRPs), such as decorin and cartilage oligomeric matrix protein (COMP), amongst others [7,8]. The functions of these matrix proteins are to permit gliding of fibril bundles and viscoelastic responses to load, provide secondary crosslinking, assist with collagen fibril organization, and provide space for proprioceptive neural networks and vascular supply. One of the largest molecular differences between tendons and ligaments is their elastin content; human tendon is 2% elastin (dry wt.) [1], but in ligament can range between 5%, 7.3% or 47% (dry wt.) in the human anterior cruciate ligament [9], posterior longitudinal ligament [2] and ligamentum flavum [10], respectively. Another difference is that tendon may have up to 34% higher pyridinoline content, a mature collagen crosslinker, compared to ligament (normalized to collagen content) [3].

T/L cells are circumferentially oriented around collagen fibers and are responsible for maintaining the density and composition of the T/L matrix. By convention, fibroblastic cells in tendon are known as "tenocytes", whereas in ligament they are known as "ligament fibroblasts". T/L cells communicate with each other using extended cell processes that terminate in connexin 32 and 43-positive gap junctions [11], and have a small multipotent stem cell population [12]. In areas of high compressive force, such as in wrap-around tendons or near a bone insertion (enthesis), fibrocartilage cells are also present [13]. Although gene-based identification of T/L cells is currently incomplete, promising markers of T/L cells include the transcription factor scleraxis (Scx) [14], the transmembrane protein tenomodulin (Tnmd) [15], and the extracellular matrix (ECM) glycoprotein tenascin-C (TN-C) [16]. Unfortunately, these cell populations show very poor healing efficacy *in vivo*; animal models have demonstrated that the mechanical properties of injured T/L are not recovered even after 12 months of healing [17].

The mechanical properties of T/L are highly complex and unique owing to their hierarchical structures and complex protein compositions. The normal response to load is non-linear, anisotropic, and viscoelastic, showing over 50% stress relaxation in 120 min [18]. Elastic moduli of adult human Achilles tendon measured *in vitro *have been found to be between 0.8-1.5 GPa [19], which is comparable and slightly higher than *in vivo *ultrasound and reaction force-plate measurements at 0.87 ± 0.2 GPa [20] or 1.16 ± 0.15 GPa [21]. *In vivo *peak bulk tissue strains in these studies have been estimated to be 8.3 ± 2.1% [20] with 18% hysteresis [21]. These properties are not comparable to cadaver Achilles or patellar tendon, which typically have elastic moduli between 200-270 MPa [22]. Although no material can accurately match the stiffness, ductility, non-linearity, and viscoelastic response of native tendon, one of the major goals of T/L engineering is to combine the appropriate biodegradable scaffold with cells and cellular cues to induce ECM remodelling that closely matches the mechanical properties and biochemistry of native tendon. Researchers have investigated both natural and synthetic biomaterials, in conjunction with many cell types, for this purpose.

### Synthetic biomaterials in tendon and ligament tissue engineering

An ideal engineered T/L would contain enough starting biomaterial for immediate load bearing post-implantation, and would degrade at a rate comparable with that of developing cellular and tissue in-growth. After a period of several weeks or months the starting material, or scaffold, would be completely replaced by regenerating T/L cells and matrix. The requirement for biodegradability and fabrication into specific tendon- and ligament-like geometries has generated interest in the use of synthetic polymer materials for T/L tissue engineering [23].

Polyhydroxyesters degrade by hydrolysis and thus have been very popular for T/L tissue engineering. An early cell attachment study found that anterior cruiciate ligament (ACL)-derived fibroblasts adhered and proliferated on poly(ε-caprolactone) (PCL), PCL/poly(DL-lactide) (PLA) (50:50), and poly(DL-lactide-co-glycolide) (PLGA) (50:50) two-dimensional substrates at a rate that was not significantly less than tissue-culture plastic [24]. Composites of these materials are often fabricated to tailor degradation rates and optimize surface energies since high hydrophilicity is thought to be detrimental to cell adhesion [25]. Several studies have built upon these earlier findings and extended T/L cell culture into three dimensions (3D) with polyhydroxyester scaffolds.

**Figure 1:**
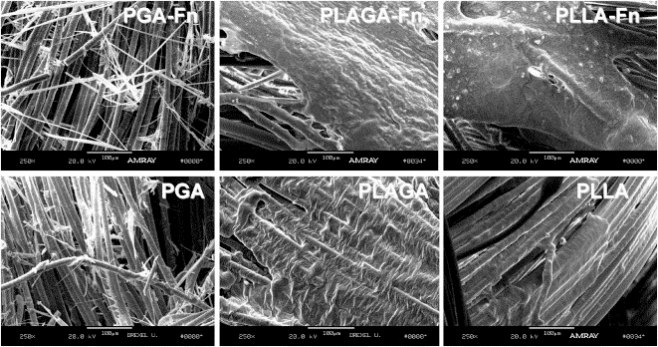
**ACL fibroblast growth and matrix formation on different synthetic braided scaffolds visualized with SEM **[26]. Images were taken after 14 days of *in vitro* culture in 10% fetal bovine serum. (Left) Culture with PGA resulted in substantial matrix degradation from acidic byproducts. (Middle) PLA and PGA in a 82:18 mass ratio showed more sustainable matrix formation, particularly with the addition of fibronectin (Fn). (Right) PLLA scaffolds also displayed considerable matrix formation, which again was amplified by the addition of Fn. Reproduced with permission from Elsevier B.V.

Lu et al. [26] fabricated 3D braided scaffolds of PGA, PLGA, and poly(L-lactic acid) (PLLA) filaments at two levels of bundling using a circular braiding loom designed to mimic the hierarchical structure of native ligament. Scaffolds were immersed in a solution of human recombinant fibronectin (Fn) to improve cell adhesion. After 14 days of culture with rabbit ACL fibroblasts, scanning electron microscopy (SEM) analysis found that cells seeded on PLLA-Fn and PLGA-Fn scaffolds produced the most matrix, and that PGA was detrimental to matrix formation, thought to be from rapidly produced acidic byproducts (Fig. 1).

Interestingly, PGA-Fn had significantly reduced cell numbers compared to PLGA-Fn and PLLA-Fn scaffolds (p < 0.05), the latter two being not significantly different from each other (p > 0.05) [26]. The authors concluded that while both the PLLA-Fn and PLGA-Fn scaffolds would be effective for ligament tissue engineering, that the PLLA-Fn scaffolds may be the more appropriate choice due to their slower degradation rate. A study of the tensile properties of braided PLGA 10:90 scaffolds found ultimate tensile strengths between 100-400 MPa, and with a circular braiding scheme, a maximum load of over 900 N, both of which were considered practical and safe for initial implantation in a human ACL replacement surgery [27]. Recent work has also elucidated ideal braiding angles of these scaffolds (60-72°) to more accurately mimic the non-linear stress-strain relationship of native T/L [28]. Taken together, these studies show great promise for future use of braided PLGA scaffolds for T/L replacement, particularly if the degradation profiles are optimally balanced against tissue ingrowth using an animal model.

Synthetic PLGA 10:90 scaffolds have also been co-fabricated with biopolymers such as collagen type I to form porous, rolled microsponges, to combine mechanical strength and cellular binding affinity for T/L tissue engineering [29]. To fabricate the microsponges, PLGA was immersed in a solution of bovine collagen type I, freeze-dried and crosslinked with glutaraldehyde. Canine ACL fibroblasts were cultured on the scaffolds for 16 days *in vitro *before rolling and subcutaneous implantation in nude mice. Using hematoxylin and eosin (H&E) staining, coupled with SEM analysis, the authors found that cells were viable and produced a uniform matrix in the scaffold center even after 12 weeks of implantation. This was a very encouraging result since the scaffolds had millimeter-scale diameters, much greater than the oft-cited O_2 _diffusion limit of 200-300 μm.

One of the advantages of synthetic polymers is that they can be processed relatively easily into fibers with nanometer-scale diameters - on the order of native collagen fibrils - using electrospinning [30]. Lee et al. [31] produced aligned polyurethane (PU) nanofiber scaffolds with average fiber diameter of 650 nm and 82% porosity using an electrospinning apparatus and a rotating collector target. Seeded human ligament cells were cultured for 48 hours then subjected to 5% uniaxial strain at 0.083 Hz using vacuum flexion on a silicone membrane for an additional 24 hours. Aligned scaffolds produced significantly more collagen mass (per DNA mass) compared to randomly oriented scaffolds (p < 0.05), and aligned scaffolds showed cell morphologies that better resembled *in vivo *morphology. This study was one of several demonstrating the superiority of aligned, as opposed to random, nanofiber orientations for T/L tissue engineering. Composite nano- and micro-fiber scaffolds have also been fabricated for T/L tissue engineering [32]. In this study electrospun PLGA (65:35) nanofibers (300-900 nm dia.) were deposited onto a mesh of 25 μm diameter PLGA (10:90) microfibers resulting in ~2-50 μm pores. The rationale for this system was that the microfibers would provide mechanical strength and degradation resistance, and the nanofibers would provide hydrophilicity and a very high surface area for cell attachment. The authors seeded porcine bone-marrow derived mesenchymal stem cells (BMSCs) and measured an average collagen production of 1.55 ng/cell after 7 days which was considered a high level of matrix production relative to other scaffold geometries. Furthermore, mRNA expression levels of relevant T/L genes, such as collagen type I, decorin, and biglycan (relative to the GAPDH), were all slightly upregulated (5-20%), suggesting partial differentiation into T/L lineages. A follow-up study reports further optimization of these scaffolds including enhanced cell attachment and proliferation using PCL and collagen type I surface deposition, and also a ~50% increased failure load by incorporating a right-angle fiber weaving method [33].

These studies and others [34,35] demonstrate some of the advantages imparted by synthetic nanofiber technologies for T/L tissue engineering, which will continue to expand and evolve as more specific T/L cell-nanofiber interactions are elucidated. At present, it appears that most studies show results favoring aligned nanofibers over randomly oriented fibers, likely due to their resemblance to native T/L fibril orientation, but the ideal fiber materials, diameters, spacing, and angles remain unresolved for T/L tissue engineering. Although single materials are often used, one promising strategy may to be incorporate fibers of different diameters and mechanical properties into a single aligned scaffold in an effort to mimic the multi-protein matrix of T/L, e.g. collagen type I, collagen type III, elastin, and proteoglycans. The rapid degradation rate of synthetic nanofibers must also be controlled and perhaps decelerated before *in vivo *implantation will be feasible. Overall, however, current synthetic nanofiber scaffolds are cytocompatible and have tailorable diameters and degrees of alignment, and are one of the most exciting prospects for the design of engineered T/L tissues.

### Natural polymeric biomaterials in tendon and ligament tissue engineering

We have seen that synthetic polymeric biomaterials have reproducible mechanical and chemical properties, are easily fabricated into different shapes and sizes, can degrade by hydrolysis, and are efficacious for T/L engineering research. However, they may lack functional chemical groups for cellular binding, and furthermore they may release acidic byproducts or unnatural polyesters into the bloodstream during degradation. For these reasons there has been considerable interest in the application of natural, protein-based fiber materials as scaffolds for T/L tissue engineering [36-40]. The most direct and obvious choice for this material is collagen type I because of its prevalence in T/L tissues. Unfortunately, no method yet exists to organize and crosslink collagen fibers in a cytocompatible manner, and as such collagen gels remain exceptionally weak with typical elastic moduli between 10-30 kPa and ultimate tensile strengths between 5-10 kPa [41]. Thus collagen-based scaffolds, while useful to investigate mechanisms of tendon differentiation and regeneration [42] and the effects of mechanical stimulation [43,44], at present are limited for T/L replacement.

Silk fibroin, rather than collagen, is a popular natural polymeric biomaterial used for T/L tissue engineering. Silk fibroin is one of two proteins excreted by *Bombyx mori *silkworms during cocoon production and is typically isolated from its sister protein sericin using sodium carbonate, urea, and/or detergents, and near-boiling temperatures [45]. *B. mori *silk fibroin is 70-80% by mass of the silk bicomplex [46], contains a heavy (350 kDa) and light chain unit (25 kDa), and is held together by the sticky cytotoxic sericin protein. The principal advantage of silk is its remarkable tensile strength and toughness (area under stress-strain curve) which is unmatched for natural proteins. Reported tensile mechanical properties of *B. Mori *silk fibroin range between 5-9 GPa for elastic modulus, 250-400 MPa for tensile strength, and 23-26% for failure strain [47]. The protein also displays surface amino acids for cell adhesion, remains structurally whole in aqueous solutions but slowly degrades (weeks-months) proteolytically *in vivo *[48,49], and can be fabricated into gels, films, braided fibers or nanofibers. These characteristics make silk fibroin one of the best natural polymers for support of cellular and tissue ingrowth for T/L tissue engineering.

Altman et al. were the first to design and test a braided silk fibroin scaffold seeded with bMSCs for T/L engineering [50]. A braided geometry with four levels of bundles twisted with 0.5 cm pitch was chosen to effectively reduce the stiffness of a single fiber to better mimic native ACL mechanical properties. A fatigue analysis with a 400 N cyclic load indicated a matrix life (after linear extrapolation) of 3.3 million cycles, expected to far outlast *in vivo *degradation. After 14 days of *in vitro *culture with human bMSCs, the authors found that cell number increased 5-fold, and that considerable matrix had been deposited. Furthermore, mRNA analysis demonstrated expression levels of T/L genes that were comparable to those in native human ACLs, such as an average collagen type I to type III ratio of ~9, an absence of collagen type II and bone sialoprotein (which would indicate cartilage or bone differentiation, respectively), and baseline expression of the T/L marker tenascin-C. The success of this initial work was considerable and highlighted the potential applicability of silk fibroin that has been properly processed and organized for *in vivo *T/L replacement.

Scaffold geometries other than braided structures have also been fabricated with silk fibroin. Liu et al. [51] surmised from previous studies of synthetic braided ligaments that such braided structures may have limited nutrient diffusion and tissue infiltration, particularly towards the center of the radial axis. To circumvent this potential issue, the authors fabricated a silk fibroin hybrid scaffold with geometries at two levels: a knitted scaffold and an interspersed microporous silk sponge. The knitted scaffold was fabricated using a 40-needle knitting machine, and the sponge was added by immersing the knitted scaffold in a low concentration silk solution, freeze drying to form pores, and then immersing in a methanol solution to prevent resolubilization. Compared with knitted scaffolds alone after 14 days of hMSC culture, the knitted-sponge scaffolds showed significantly higher biological responses with nearly every evaluation method (p < 0.05), including cellular proliferation, GAG production, viable cell density, mRNA expression of collagen types I and III, and tenascin-C, and collagen-based matrix production, confirming the positive benefit of the microporous silk sponge. However, no significant differences in maximum tensile load or stiffness were recorded compared to unseeded scaffolds after 14 days, suggesting that the secreted matrix did not contribute towards scaffold strength. Also, the tensile strength and stiffness were far below (<20%) those of the adult human ACL [52], which was their target tissue. Nevertheless, the cytocompatability and rapid T/L-like matrix development is impressive and further demonstrates the effectiveness of a cyto-friendly composite structure.

**Figure 2:**
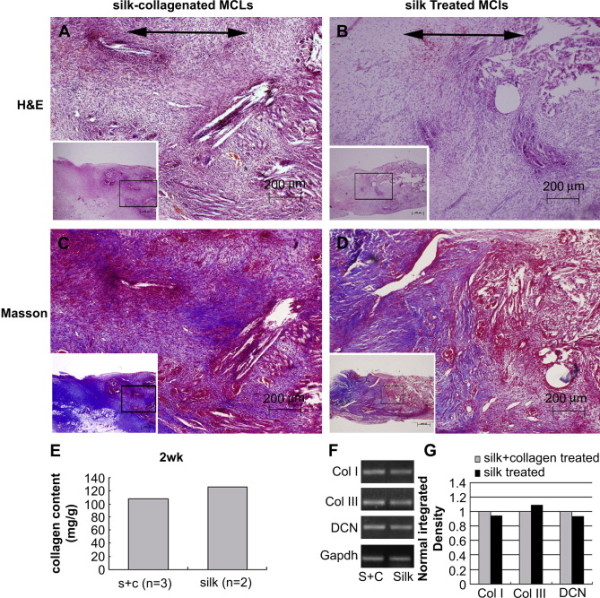
**Histological and gene expression analysis of the extent of *in vivo *repair after 14 days using silk and silk/collagen scaffolds in a rabbit model of MCL transection **[53]. (A, B) H&E staining showed relatively unorganized matrix formation but a high cell density in silk/collagen scaffolds than silk alone. (C, D) Masson's trichrome staining revealed the beginnings of collagen fiber formation (indicated by blue areas), and more collagen deposition in the silk/collagen scaffold group. (E) Total collagen content was reduced in the silk/collagen group (S + C), but, as detected by RT-PCR shown in (F, G), this could have been due to a reduction in the expression of collagen type III, an alternative collagen type for the ligamentous phenotype than collagen type I and decorin, both of which showing higher level of expression in the silk/collagen scaffold group. Reproduced with permission from Elsevier B.V.

In addition to composite synthetic scaffolds, composite natural scaffolds have also been fabricated for T/L tissue engineering applications [53]. In this work a knitted silk fibroin base matrix was infiltrated with a freeze-dried collagen type I microsponge. MSC-seeded scaffolds were implanted into a rabbit medial cruciate ligament (MCL) transection model to evaluate *in vivo *repair potential over 12 weeks. *In vitro*, the authors found that gene expression levels of T/L-associated genes by cells seeded in silk-collagen scaffolds compared to silk alone were substantially higher, and showed collagen type I elevated by 250% and decorin elevated by over 500%. Furthermore, histological analysis with H&E and Masson's trichrome staining of the repairing MCL found more tissue ingrowth compared to silk alone and untreated MCLs after 2, 4, and 12 weeks (Fig. 2). These results suggest that a chimeric silk-collagen sponge matrix may be an effective treatment for MCL transections due to its rapid tissue ingrowth and favorable genetic expression results. Optimization of sponge pore size may be an important advancement in the development of these engineered T/L scaffolds.

One of the most important evaluation tests in T/L engineering is the success of an engineered graft to be regenerated by the body into functional T/L. Recent long-term studies by Fan et al. have investigated silk scaffold T/L regeneration in rabbit [54] and pig models [55], providing new insights into the kinetics of T/L tissue ingrowth and scaffold degradation. First, a rabbit ACL reconstruction model [54] was tested by transecting healthy rabbit ACLs and surgically implanting an MSC-seeded knitted silk scaffold with microporous silk sponge into bone tunnels of rabbit knees. A morphological and histological evaluation of implanted silk grafts replacing the rabbit ACL after 8 weeks suggested substantial production of collagen type I, collagen type III, and tenascin-C, and that the ligament-bone attachment was stable (evaluated with micro-CT). However, similar to other studies with knitted silk scaffolds the tensile strength and stiffness were unfortunately well below those of native human ACL. A subsequent study using the larger porcine model showed similar, encouraging results. To compensate for the additional load bearing, the knitted-sponge silk scaffold was rolled around a braided silk cord and again seeded with MSCs. After 24 weeks the gross morphological and histological characteristics were evaluated and as before, collagen type I, collagen type III, and tenascin-C were all distinctly present as shown by immunohistochemistry analysis. However, with an average failure load of 398 N, the authors noted that regenerated scaffolds at 24 weeks could be effective for mild daily load bearing, but would likely not survive trauma or vigorous exercise. Unfortunately the scaffold strength at earlier time points was not measured; it is clear however that 24 weeks would likely be too long for most patients to wait before mild load bearing would be feasible. Nevertheless the clinical implications of this study are profound, and demonstrate the *in vivo *effectiveness of a multi-structural silk scaffold for T/L tissue engineering.

Because of their inherent biocompatibility, natural polymeric macromolecules will remain at the forefront of biomaterials research for T/L tissue engineering. Although collagen type I gels may eventually be the scaffold material to use, their ultimate tensile strengths and elastic moduli are currently too low to act as a load bearing material. In the interim, silk fibroin has emerged as an excellent natural biomaterial alternative to collagen and has already been shown to regenerate T/L in large scale animal models. Assuming the success of longer-term animal trials, it is anticipated that impending clinical trials with silk fibroin-based engineered ligaments will confirm the efficacy of silk to restore T/L function after injury and offer an exciting new option for T/L repair.

### Advanced scaffolds and signaling factors in ligament tissue engineering

#### A. Functionalized T/L scaffolds

One of the key factors in effective application of material scaffolds in tissue engineering is the optimization of cell-biomaterial interactions, particularly in terms of the ability of cells to adhere, proliferate, and secrete matrix onto the scaffold. Synthetic and natural polymers are effectively long chains of repeating chemical units, and it is thus possible to link small molecules covalently to their surfaces to enhance cell adhesion, proliferation, and matrix production. Such an approach has high potential, since biomaterials that natively lack chemical cell attachment groups can also be functionalized, thus expanding the range of implantable biomaterials. Cell-matrix interactions are typically mediated via cell surface integrin receptors, which are specialized transmembrane proteins that are connected cytoplasmically to the actin cytoskeleton. The most common example of an integrin-interacting matrix epitope is the peptide sequence, RGD (arginine-glycine-aspartic acid) [56], which has been used to functionalize a number of biomaterials.

The practice of functionalizing grafts to improve engineered tissues is sometimes done passively by merging one scaffold with another, for the purpose of combining mechanical properties with integrin binding capability. An example of passive functionalization is a recent work by Garcia-Fuentes et al. [57] who blended hyaluronan, a common native glycosaminoglycan, with silk fibroin and seeded MSCs for general regenerative applications. Matsumura et al. [58] conducted a more direct functionalization study and modified poly(ethylene-co-vinyl alcohol) (PEVA) films with carboxyl groups (COOH) and subsequently covalently attached collagen type I, designed to enhance periodontal ligament adhesion to PEVA-coated titanium dental implants. Other general tissue engineering applications have used a variety of functionalizing groups including phosphate, amide and sulfonate groups.

There has been some interest in adding functional groups to non-degradable synthetic graft surfaces in the hopes of enabling tissue growth and avoiding poor tissue integration, foreign body immune responses, and high failure rates. Zhou et al. [59] recently functionalized polyethylene terephthalate (PET) grafts with poly(sodium styrene sulfonate) (PNaSS) functional groups and observed fibroblastic cell response. These investigators functionalized PET by first exposing PET fabrics to ozone gas (O_3_), which is unstable and breaks into O_2 _and O•, the latter of which transfers its free radical to the PET surface making it much more reactive. Under an inert argon atmosphere, the samples were then immersed in a bath of monomer 15% (w/v) sodium *p*-(styrene sulfonate) (NaSS) at 65-70°C temperature, forming polyNaSS on the PET surface by radical polymerization. The human fibroblast McCoy cell line was seeded onto the functionalized PET surface and observed after 4 days of culture with the live-cell fluorescence label calcein AM. Captured images revealed considerably more cell adherence onto functionalized fibers than non-functionalized fibers. Additionally, dynamic fluid testing indicated that cells adhered to polyNaSS-PET scaffolds compared to PET alone required 12-fold more shear stress for 50% of adhered cells to be removed (12 dyn/cm^2 ^compared to 1 dyn/cm^2^). The authors attributed these profound results to two factors: the enhanced surface hydrophilicity enabling cell spreading, and the opportunity for fibronectin to bind to the PNaSS, increasing the number of focal adhesion contacts and the potential for organized cytoskeletal formation. It will be particularly interesting when these functionalized grafts are implanted *in vivo *to test if the foreign body capsulation response still occurs, and if not, how they perform over long term implantation.

#### B. Decellularized T/L scaffolds

It has been argued that the best replacement biomaterials for T/L are those derived from the T/L themselves (when they are available) because the tissues already have similar mechanical properties and because the endogenous integrin binding sites are present and abundant in the native ECM. However, some studies have suggested that allograft tissues can contain residual donor cells even with strict sterilization and cleaning [60], and may cause a significant inflammatory response when implanted *in vivo *[61]. Furthermore, there are concerns about the extent and efficiency of cellular infiltration particularly to the dense center areas of the graft. Thus, the application of chemical treatments to yield a fully decellularized and more porous scaffold, as opposed to the use of minimally treated allografts [62], is preferred for T/L tissue engineering. Whitlock et al. [63] recently addressed this issue with a novel oxidative chemical treatment and a battery of *in vitro *cytocompatibility and tissue tests. The authors isolated adult chicken flexor digitorum profundus tendons and added 1.5% peracetic acid to act as an oxidizing agent (using OH radicals) to create pores in the tissue and remove loose DNA. Simultaneously, the detergent Triton X-100 (polyethylene glycol octylphenyl ether) was also added at 2% concentration to lyse cell membranes. When compared to non-treated controls, the oxidized scaffolds had no nuclei visibly present (H&E, DAPI staining) and at minimum 70% less total DNA, which was considered a promising result. The scaffolds also appeared more porous, and on average had 25% less elastic modulus and stiffness (p > 0.05). Results from a subcutaneous rat *in vivo *cell infiltration study showed that after 3 weeks, cells with nuclei were present in the outside layers and some inner layers of the scaffold, and that no inflammatory reaction or capsule formation was present. Future studies with human T/L allografts utilizing a combination of a lysing agent and oxidiative agent are warranted, and these treatments may prove to be effective in minimizing the foreign body and capsulation responses found with standard allografts.

#### C. Effects of growth factors on natural and engineered tendons and ligaments

One of the most promising strategies to augment natural regeneration is the introduction of growth factors with specific activities on target tissues. Of the many growth factors in the body, the following five growth factors have shown promise for T/L engineering due to their notable upregulation during T/L healing [64]: insulin-like growth factor-I (IGF-I), transforming growth factor-β (TGF-β), vascular endothelial growth factor (VEGF), platelet-derived growth factor (PDGF), and basic fibroblastic growth factor (bFGF). Once considered impractical due to their extremely high costs, growth factors have recently been re-evaluated for their possible application for T/L tissue engineering [65-68], made possible by biotechnological advancements in the production and purification of recombinant proteins. Of these five growth factors, bFGF has shown particular efficacy and has been known as an effective promoter of T/L regeneration since early wound healing studies of canine dental defects [69], and is now beginning to be incorporated into full T/L tissue engineering strategies. Sahoo et al. [70] blended bFGF with PLGA and produced electrospun nanofibrous scaffolds capable of releasing 60% of the growth factor over the course of one week. When these scaffolds were seeded with bone-marrow derived rabbit MSCs and compared against scaffolds without bFGF, the authors found significant increases in cell proliferation, immunostaining of tenascin-C and collagen types I and III, and gene expression levels of collagen types I and III, fibronectin, and biglycan over 14 days, which are all promising indicators of enhanced T/L differentiation. Impressively, these results were attained with a bFGF concentration of only 11 μg/mL of PLGA (dissolved in hexafluoroisopropanol). Recently, bFGF was also employed *in vivo *in a rabbit ACL repair model via loading into gelatin hydrogels to form a three-part engineered ligament consisting of a braided fibronectin-coated PLLA core, a collagen membrane wrapping sheet, and the bFGF-loaded gelatin hydrogels [71]. Observation at 8 weeks post-implantation revealed that the addition of bFGF increased both the maximal strength and stiffness of the regenerated ACL by approximately 50%, increased collagen mass in the regenerated tissue by 2.5-4 fold, and produced histological cross-sections that more closely resembled native ligament. This study demonstrates that local and controlled release of growth factors such as bFGF can be potent accelerators of T/L regeneration *in vivo*.

**Figure 3:**
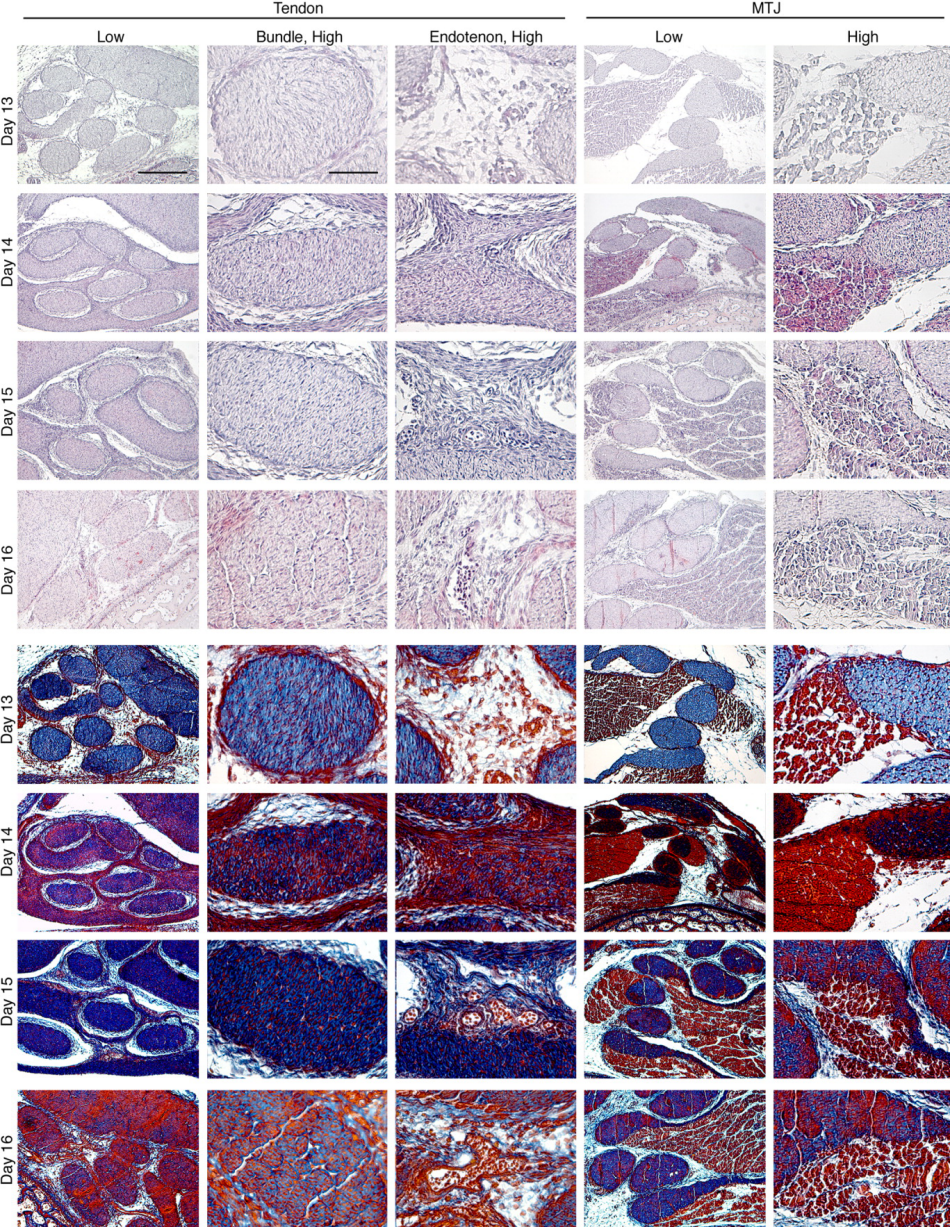
**Histology of transverse sections of intermediate tendon of chick embryos at developmental days 13, 14, 15, and 16 **[72]. (Top) Hematoxylin-eosin staining; (Bottom) Mallory's trichrome staining. Low magnification, Bar = 200 μm; high magnification, Bar = 50 μm.

In addition to bFGF, TGF-β has also been implicated as an important growth factor in T/L development, and is actively being investigated for applications in T/L tissue engineering. We characterized the spatiotemporal distribution of TGF-β in the developing chick tendon during embryonic days 13-16 (Fig. 3; [72]). Histologic results demonstrated rapid tissue organization and development during this time period. Immunohistochemical staining showed TGF-β2 and -β3, but not TGF-β1, were present within the tendon mid-substance on all days studied. TGF-β2 and -β3 exhibited similar distribution patterns, but differed in timing and intensity. Taken together, these findings strongly support the postulate that TGF-β2 and -β3 are involved in tendon development and that these isoforms may have independent roles. This was recently confirmed when TGF-β2 and -β3 knockout mice were unable to form most tendon and ligaments, and both TGF-β2 and -β3 were shown to be essential for maintenance of tendon progenitor cells *in vivo*[73]. In the near future, as production of purified growth factor continues to become more economical, more research on regenerative growth factors will be performed, leading to information that will guide the use of growth factors as integral components of functional T/L replacement tissues.

#### D. Effects of cyclic strain on engineered ligaments

**Figure 4:**
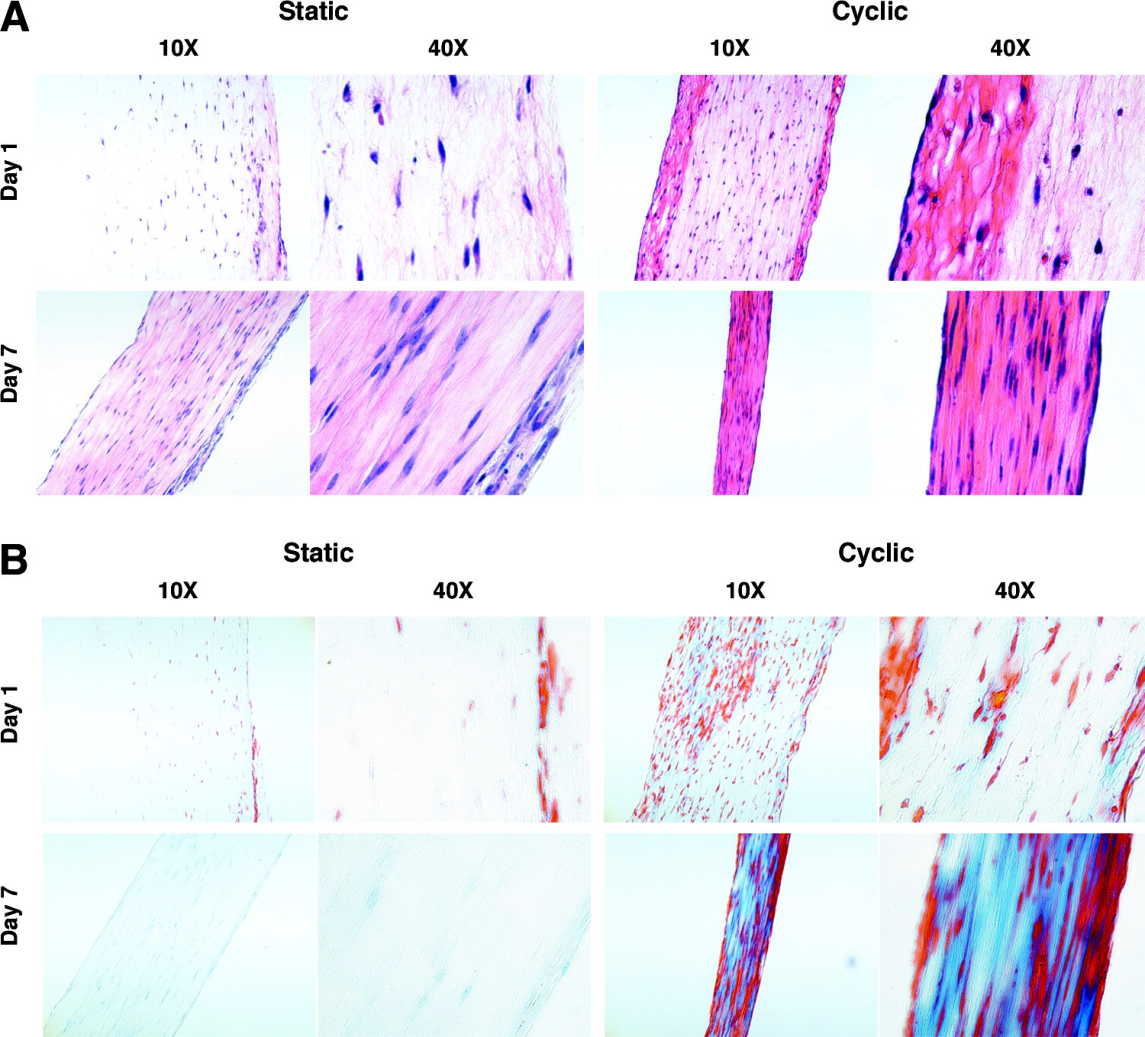
**Histology of MSC-collagen constructs harvested after 1 and 7 days of static and cyclic loading **[85]. Longitudinal 5-μm-thick sections histologically stained with Mallory's trichrome. A higher level of matrix staining is seen consistently in constructs subjected to cyclic loading.

It is well known that locomotion induces tensile strain on T/L tissues, and for more than 100 years [74] it has been theorized and later demonstrated that the density and remodeling of bone is related to its loading state [75,76]. Thus it should be no surprise that since nearly the inception of the field, tissue engineers have studied the effect of forces on fibroblasts [77] and later on ACL cells [78]. Subsequent to these preliminary experiments, more elaborate bioreactors with uniaxial or multi-axial applied forces were developed for musculoskeletal tissue engineering. Altman et al. [79] developed a novel cyclic strain bioreactor that simultaneously applied 10% tensile strain and 25% torsional strain to MSC-seeded collagen type I gels, designed to mimic the natural 90° twist on ACL collagen fibers during knee flexion/extension. The stimulation was applied at a rate of 0.0167 Hz for up to 21 days. Compared to static construct controls, the mechanically stimulated gels had significantly higher cross-sectional cell density and a 2.5-fold increase in cell alignment. The most striking finding was that the mRNA expression of collagen type I, collagen type III, and tenascin-C in mechanically stimulated gels were all significantly upregulated compared to static controls and approached native T/L expression, and that bone and cartilage markers were not upregulated. This was the first study to demonstrate that MSCs could begin to be differentiated into T/L-like lineages using mechanical stimulation alone. The application of cyclic strain in stem cell based bioreactor systems to promote cell proliferation, cell alignment, and T/L-marker expression is now an accepted and widely utilized method in T/L tissue engineering [80]. This and other studies have demonstrated enhanced matrix production by MSCs when cyclically loaded under uniaxial tension in long-term cultures, but have not elucidated the mechanisms for these results [81-84]. To investigate potential mechanisms, we conducted short term studies with human MSCs in a similar model system utilizing collagen type I scaffolds and uniaxial tensile loading, and investigated putative tendon marker expression [85]. Results showed that while static uniaxial tensile loads upregulated scleraxis expression, cyclic loading significantly enhanced collagen matrix production (Fig. 4). Cyclic loading was necessary to sustain mRNA levels of scleraxis, a tendon-specific marker gene, and differentially regulated additional developmental and mature tendon marker molecules, including collagens, Wnts and MMPs. The results of this study supported the premise that dynamic mechanical loading enhances tenogenesis of hMSCs and provided insights into the mechanisms of this process.

Synthetic biomaterials have also been implemented in cyclic strain bioreactors for T/L tissue engineering. Moe et al. [86] seeded human dermal fibroblasts on PLGA 10:90 knitted scaffolds and applied 1.8% tensile strain for 4 hours daily over two weeks at either static, 0.1 Hz or 1 Hz strain rates. Using H&E staining the authors found the most substantial cellular alignment with a 0.1 Hz applied strain rate; however, the scaffold stiffness of mechanically stimulated constructs was significantly less than that of static controls. However, the stiffness was not normalized to cross-sectional area (i.e. elastic modulus), and it is thus difficult to know if cell contraction and/or matrix formation were partially responsible for this result. Raif and Seedhom [87] also seeded cells onto a knitted scaffold, but used non-degradable PET fibers and bovine synovial cells, which appear to have the capacity to be de-differentiated into multipotent cells [88]. The applied strain parameters varied considerably between 0.65-4.5% strain magnitude at 1 Hz and was applied for 1-4 hours per day for either 1 or 35 days. The authors found that cell proliferation during short-term application was reduced at 1 hour post-stimulation, but was upregulated 22 hours later. Furthermore, cell proliferation increased as cyclic strain amplitude was increased, suggesting a higher affinity for differentiation or matrix production at lower strain amplitude, or simply less cell proliferation. The long term (35-day) study did not find significant differences except that higher strains tended to result in a higher scaffold cellular density.

**Figure 5:**
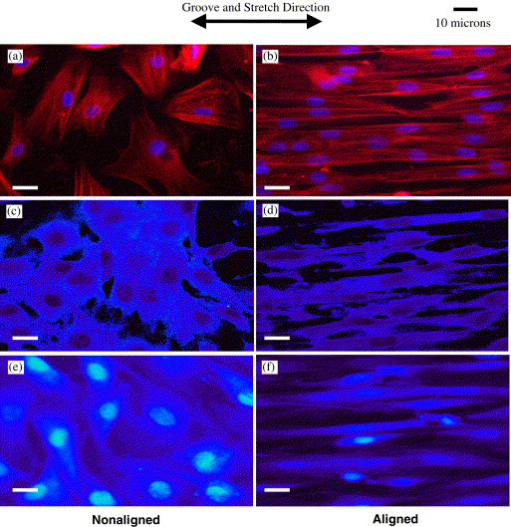
**Fluorescence images of actin filaments (red) and nuclei (blue) of MCL fibroblasts cultured on collagen-coated PDMS surfaces **[90]. (Left column) Cells cultured on smooth PDMS substrates showed randomly aligned cytoskeletal and nuclear morphology, even after 3.5% cyclic strain at 1 Hz for 2 hours (c). (Right column) Cells cultured in PDMS microgrooves with 10 μm width and 6 μm depth were substantially more aligned, based on cytoskeletal and nuclear morphology, compared to smooth PDMS, especially after being cyclically strained (d). (e, f) Control cells grown in the custom cyclic strain incubator without strain applied showed similar results to cells cultured in a standard incubator (a, b). Reproduced with permission from Elsevier B.V.

In addition to mechanical stimulation, recent studies have investigated the combinatorial effect of mechanical stimulation and cellular alignment. The developing underlying theory of the influence of patterned scaffold structures is known as 'contact guidance', which states that cellular response, especially alignment and proliferation, is dependent on the size and type of the channeling structure. Mechanically stimulating ACL cells seeded on polydimethylsiloxane (PDMS) micropatterned surfaces with an applied 8% uniaxial strain at 0.5 Hz for 4 hours a day and 2 days revealed the expression of several novel genes influenced by mechanical stimulation [89]. Specifically, microarray-based real-time PCR analysis showed that expression of the following genes decreased during normal culture but increased after mechanical stimulation: MGP (matrix Gla-protein, 3.8-fold), GADD45A (growth arrest and DNA damage-inducible gene, 2.3-fold), UNC5B (unc-5 homolog B, 1.6-fold), TGFB1 (transforming growth factor-β1, 1.4-fold), COL4A1 (collagen type IV α1, 1.2-fold), and COL4A2 (collagen type IV α2, 1.2-fold). The authors noted that MGP is a small matrix protein that may be involved in cellular differentiation, and that GADD45A may influence cell cycle proteins and play a role in genomic stability. The exact functions of some of these genes in T/L biology are not well understood and certainly warrant further investigation, especially with other cell types. In a similar study, Jones et al. [90] seeded rat MCL cells on a microgrooved PDMS substrate and applied 3.5% strain at 1 Hz for 2 hours. The authors found considerably more alignment in the groove and stretch direction compared to cells grown on a smooth PDMS surface (Fig. 5), and that intercellular propagation of mechanically induced Ca^2+ ^flux was significantly enhanced with the application of cyclic strain (p < 0.001), but not when grooves alone were introduced. Taken together, these findings suggest that uniaxial mechanical stimulation and not only forced cellular alignment is necessary to produce positive benefits for T/L tissue engineering.

While mechanical stimulation is a relatively new practice in tissue engineering, it is actively being employed to develop a variety of musculoskeletal engineered tissues, including tendon, ligament, muscle, and bone constructs. In most if not all cases, the addition of cyclic strain has conferred a positive benefit especially with cell proliferation, density, and differentiation of stem cells towards musculoskeletal lineages. However, one important admonition is that the ideal mechanical stimulation regime is far from being described, and specific biological models of mechanical stimulation to tissues are lacking. Thus, given the excellent cost-benefit ratio of applying mechanical stimulation, there is a need for further optimization of applied tissue strains with T/L tissue systems, and perhaps in the future applying an optimized mechanical stimulation regime to engineered T/L will be commonplace.

## Conclusions

The field of T/L tissue engineering is progressing at an increasingly rapid pace; in just 10 years engineered tendons and ligaments have advanced from concept to capable of regenerating large animal ligaments in long term studies. The potential health care implications of engineered T/L are extensive and with an aging population will become more important with time.

The design factors for T/L engineering have thus far included: native T/L anatomy, biomaterial mechanical properties, biomaterial degradation rate, cellular adherence/spreading on biomaterials, and matrix formation. From a biomaterials perspective, it is clear that matching biomaterial properties to the native T/L structure and function is a critical consideration. Yet what has also been important for the progression of the field has been the utilization of combinatorial approaches; examples of this include merging braided scaffolds with sponges, merging two materials into a single scaffold, functionalizing a biodegradable surface, or adding mechanical stimulation to aligned cells. In all cases there is not one superior engineered T/L design, and thus far the ideal engineered tendon or ligament has yet to be created. Some of the important future milestones of T/L tissue engineering include improving the strength and biological integrity of the tendon-muscle and tendon/ligament-bone junctions of implanted engineered T/L, developing scaffolds and models that match the rate of scaffold degradation with the rate of tissue ingrowth, matching native T/L elastic and viscoelastic mechanical properties, and developing T/L disease models through tissue engineering. Meeting the demands of these requirements will require concentrated interdisciplinary efforts from biologists, chemists, biomaterials scientists and tissue engineers, which will eventually provide a new and improved option for repair of injured tendons and ligaments to thousands of patients in need of help.

## Competing interests

The authors declare that they have no competing interests.

## Authors' contributions

CKK & JEM wrote the manuscript. RST edited and proofread the manuscript. All authors have read and approved the final manuscript.
